# Exosomes: Decreased Sensitivity of Lung Cancer A549 Cells to Cisplatin

**DOI:** 10.1371/journal.pone.0089534

**Published:** 2014-02-21

**Authors:** Xia Xiao, Shaorong Yu, Shuchun Li, Jianzhong Wu, Rong Ma, Haixia Cao, Yanliang Zhu, Jifeng Feng

**Affiliations:** 1 Research Center for Clinical Oncology, Nanjing Medical University Affiliated Cancer Hospital, Jiangsu Cancer Hospital and Jiangsu Institute of Cancer Research, Nanjing, Jiangsu Province, China; 2 Nanjing University of Technology, Nanjing, Jiangsu Province, China; 3 Southeast University, Nanjing, Jiangsu Province, China; Wayne State University School of Medicine, United States of America

## Abstract

Exosomes are small extracellular membrane vesicles of endocytic origin released by many cells that could be found in most body fluids. The main functions of exosomes are cellular communication and cellular waste clean-up. This study was conducted to determine the involvement of exosomes in the regulation of sensitivity of the lung cancer cell line A549 to cisplatin (DDP). When DDP was added to A549 cells, exosomes secretion was strengthened. Addition of the secreted exosomes to other A549 cells increased the resistance of these A549 cells to DDP. Upon exposure of A549 to DDP, the expression levels of several miRNAs and mRNAs reportedly associated with DDP sensitivity changed significantly in exosomes; these changes may mediate the resistance of A549 cells to DDP. Exosomes released by A549 cells during DDP exposure decreased the sensitivity of other A549 cells to DDP, which may be mediated by miRNAs and mRNAs exchange by exosomes via cell-to-cell communication. Although the detailed mechanism of resistance remains unclear, we believed that inhibition of exosomes formation and release might present a novel strategy for lung cancer treatment in the future.

## Introduction

Lung cancer is the leading cause of cancer-related mortality in the word and non-small-cell lung caner (NSCLC) is the most common form of lung cancer. Patients with such an aggressive tumor have a poor five-year survival rate less than 20%, which is most likely attributed to metastatic disease at the time of diagnosis. Although several target drugs such as erlotinib and cetuximab could increase the overall survival of NSCLC patients, platinum-doublet chemotherapy remains the most important treatment for patients with advanced NSCLC.

Platinum (DDP) is a DNA-damaging agent that could enter tumor cells and cause aquation and hydrolysis to form reactive platinum species [1]. Aquated DDP generally recognizes DNA as the primary target and interacts with DNA, leading to the formation of interstrand and/or intrastrand crosslinks [2]. The DNA-damage response (DDR) system and diverse signaling pathways are activated [3], and the expression levels of RNAs could be influenced accordingly [4]. Many patients display either innate insensitivity to the drug or DDP-insensitivity recurrent of the disease following an initial period of treatment. Multiple mechanisms are involved in the sensitivity regulation of tumor cells to DDP; these mechanisms include intracellular accumulation and efflux of DDP [5], DNA repair ability, tolerance to unrepaired DNA lesions [6], and regulation of several genes [7].

Exosomes are small extracellular membrane vesicles, which could be secreted by many kinds of tumor cells and exist in most body fluid [8], [9]. Regardless of origin, exosomes have similar protein compositions, which can be categorized into three major groups: genuine raft proteins, cytoskeleton like proteins and heat shock proteins [10]. Internal vesicles are formed by the inward budding of cells known as multivesicular endosomes (MVE). Fusion of the MVE with the plasma membrane leads to the release of the internal vesicles known as exosomes [11]. Exosomes could travel to surrounding cells or distant tissues to display functions such as immune stimulation, immune suppression [12], induction of proliferation and tolerance [13]–[15], transfer of genetic material [10] and garbage removal [16]. Exosomes contain a substantial amount of RNA and could be transferred from one cell to another [10], thereby contributing to the proliferation and metastasis of cancer and cancer development [13], [14], [17], [18]. However, to the best of our knowledge, the involvement of exosomes in the regulation of sensitivity of lung cancer cells to DDP remains unknown.

Once exposed to DDP, tumor cells usually adapt to the microenvironment and adjust to stimulation. Since exosomes are reported to be involved in cell communication, we hypothesized the possible involvement of exosomes in the regulation of A549 cell responses to DDP. Specifically, exosomes released by A549 cells during DDP exposure may alter the sensitivity of the surrounding cells to DDP. In addition, exosomal RNAs can be transferred from one cell to another [10]. As such, we supposed that some miRNAs and mRNAs reportedly associated with DDP resistance might be transferred from one cell to another by exosomes. MiR-21, miR-98, miR-133b, miR-138, miR-181a and miR-200c [19]–[24] were reportedly associated with DDP sensitivity regulation, whereas ERCC1, BRCA1 and RRM1 [25]–[27] were related to DDP resistance. To test our hypotheses, these miRNAs and mRNAs were selected to preliminarily study their involvement in the process of DDP resistance.

## Results

### Characterization of Exosomes Released by A549 Cells

**Figure 1:**
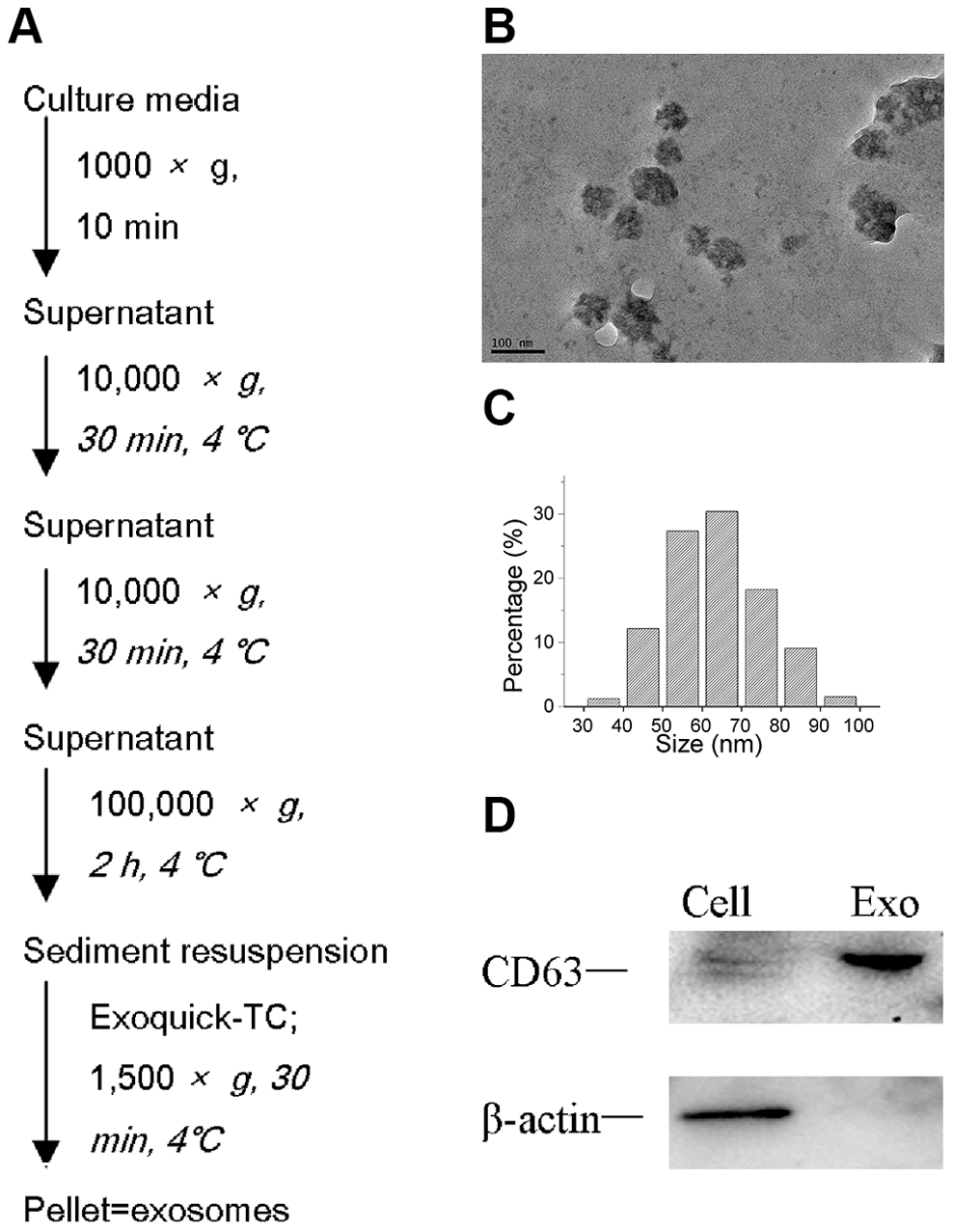
Isolation and characterization of exosomes. (A) Exosome isolation method used in this study. (B, C) Transmission electron microscopic image of the exosomes. Exosomes are small vesicles measuring from 30nm to 100 nm in diameter. (D) Western blot of CD63 and β-actin in exosomes and cells.

To ensure successful isolation of exosomes, the collected exosomes were observed by TEM (transmission electron microscope) and analyzed by Western blot (Figure 1A). Microvesicle clusters exhibited round vesicles measuring 30–100 nm in diameter (Figures 1B, 1C). Figure 1D shows the expression of CD63, a tetraspanin family member that localizes in exosomal internal vesicles, as a dual band in exosomes and as a light band in cells.

### Cellular Effect of A549 Cells to Cisplatin

**Figure 2:**
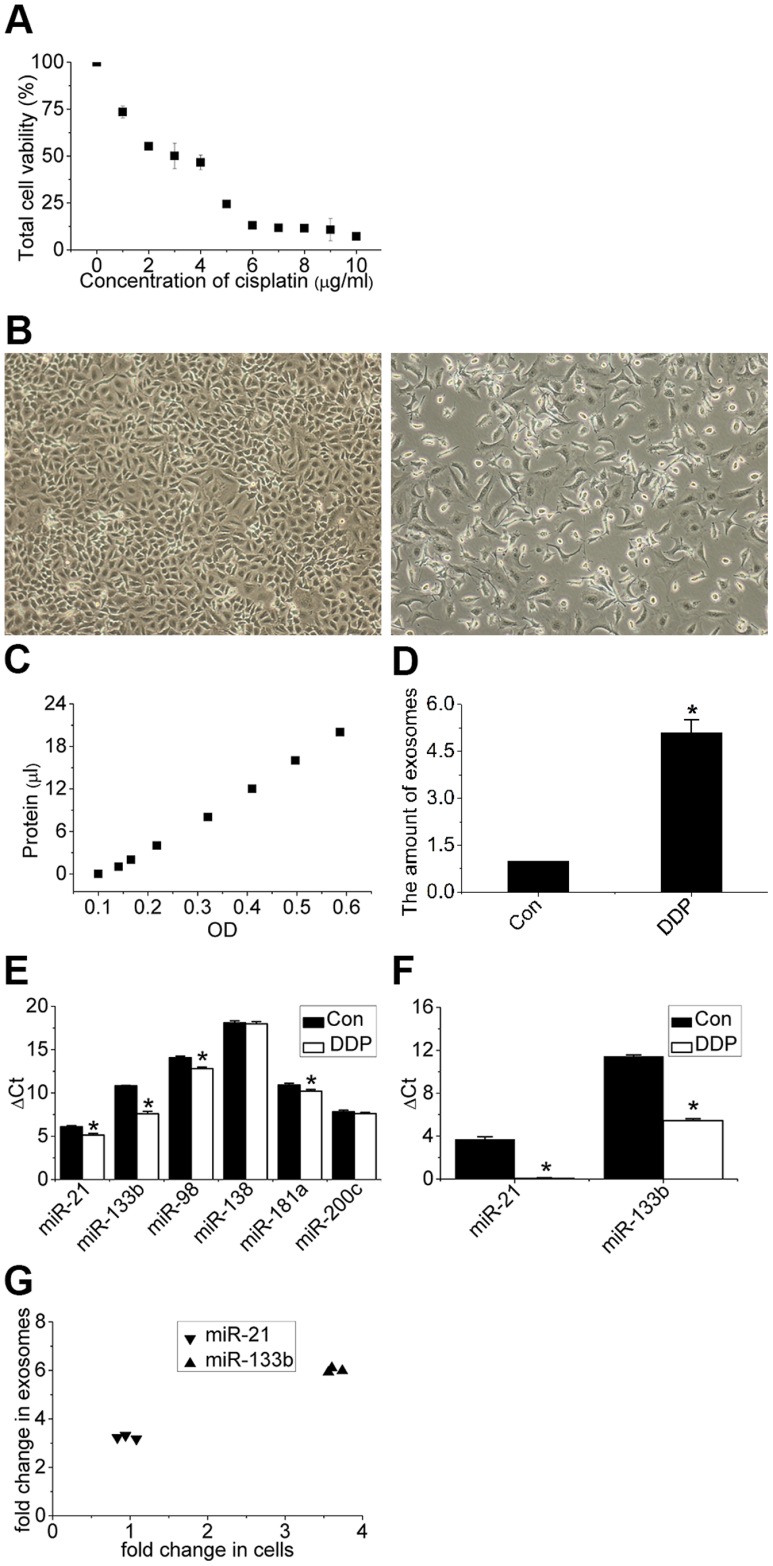
Response of A549 cells to cisplatin. (A) Dose response relationship between viability of A549 cells (%) and concentration of cisplatin (1–10 µg/mL) for 48 h. (B) Microscopic image of A549 cells treated with 0 and 3 µg/mL cisplatin. Images were obtained at a magnification of 100×. (C, D) Quantity of exosomes determined via the BCA standard curve and enhanced secretion of exosomes normalized to cell numbers after exposure of A549 cells to cisplatin. (E) Differential expression levels of the six miRNAs in cells after exposure of A549 cells to cisplatin. (F) Differential expression levels of the two miRNAs in exosomes after exposure of A549 cells to cisplatin. (G) Fold-changes in expression of miR-21 and miR-133b in exosomes are higher than those in cells. Bars indicate mean ± SD of three replicates. *indicates p<0.05 versus control groups.

Dose-response evaluation was performed in this study. A concentration of 3 µg/mL DDP was selected for our protocol since this dose caused the death of about 50% of A549 cells (Figures 2A, 2B). To test the effect of DDP on the release of exosomes, exosomes were quantified with BCA; the protein standard curve was shown in Figure 2C. As shown in Figure 2D, the amount of exosomes released by each cell during DDP exposure was higher than that under normal conditions, which indicates a significant increase in exosomes released by A549 cells upon exposure to DDP.

To determine if exosomes could shuttle information, the expressions of the six miRNAs proposed in this paper were detected in cells and exosomes. All six miRNAs could be detected in cells, whereas only miR-21 and 133b could be detected in exosomes (the Ct value of the four other miRNAs exceeded 40). To test the influence of DDP on the expression levels of miRNAs in cells and exosomes, the expression levels of these six miRNAs were detected in cells and exosomes upon exposure of A549 cells to DDP. As shown in Figures 2E and 2F, the expression levels of miR-21, miR-133b, miR-98, miR-181a increased in cells while the expressions of miR-21 and 133b increased in exosomes. Fold-changes in expression of miR-21 and miR-133b in exosomes were also higher than those in cells (Figure 2G).

### Exosomes Decrease the Sensitivity of A549 Cells to DDP

**Figure 3:**
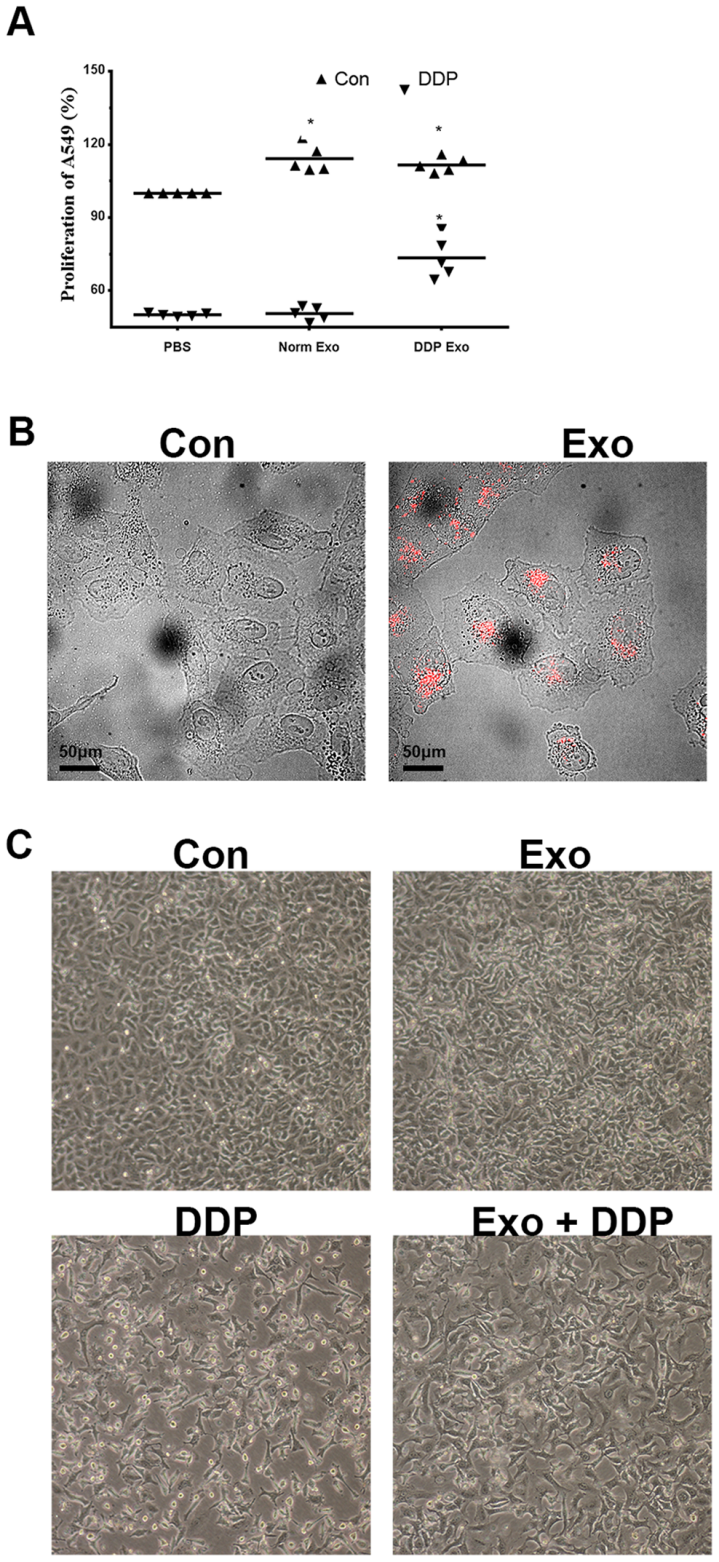
Influence of exosomes on sensitivity of cells to cisplatin. (A) Pretreatment of A549 cells with exosomes released during cisplatin (3 µg/mL) exposure decreases the sensitivity of cells to cisplatin by about 20% compared with those without pretreatment for 48 h. (B) Uptake of exosomes (red) by A549 cells after incubation for 3 h was visualized by microscopy. Scale bar: 50 µm. (C) Microscopic image of A549 cells cultured under four conditions [control, exosomes, DDP (3 µg/mL), exosomes+DDP] for 48 h. Images were obtained at a magnification of 100×. *in (A) indicates p<0.05 versus control groups.

To determine exosomes uptake by A549 cells, exosomes were labeled by DiD and co-incubated with A549 cells for 3 h, after which fluorescence microscopy was performed. As shown in Figure 3B, exosomes could be absorbed by surrounding cells. To determine whether exosomes could influence the sensitivity of A549 to DDP, exosomes released by A549 cells under normal condition and under DDP condition were harvested and added to cells. As shown in Figures 3A and 3C, exosomes released by A549 cells under DDP exposure could decrease the sensitivity of other A549 cells to DDP, while the exosomes released by A549 cells under normal conditions had little influence on the sensitivity of A549 cells to DDP. Because exosomes released by A549 cells under normal conditions did not influence the sensitivity of surrounding cells to DDP, we elected to study the function of exosomes released by A549 cells under DDP exposure only.

### Exosomes Influence the Expression Levels of Cellular miRNAs and mRNAs

**Figure 4:**
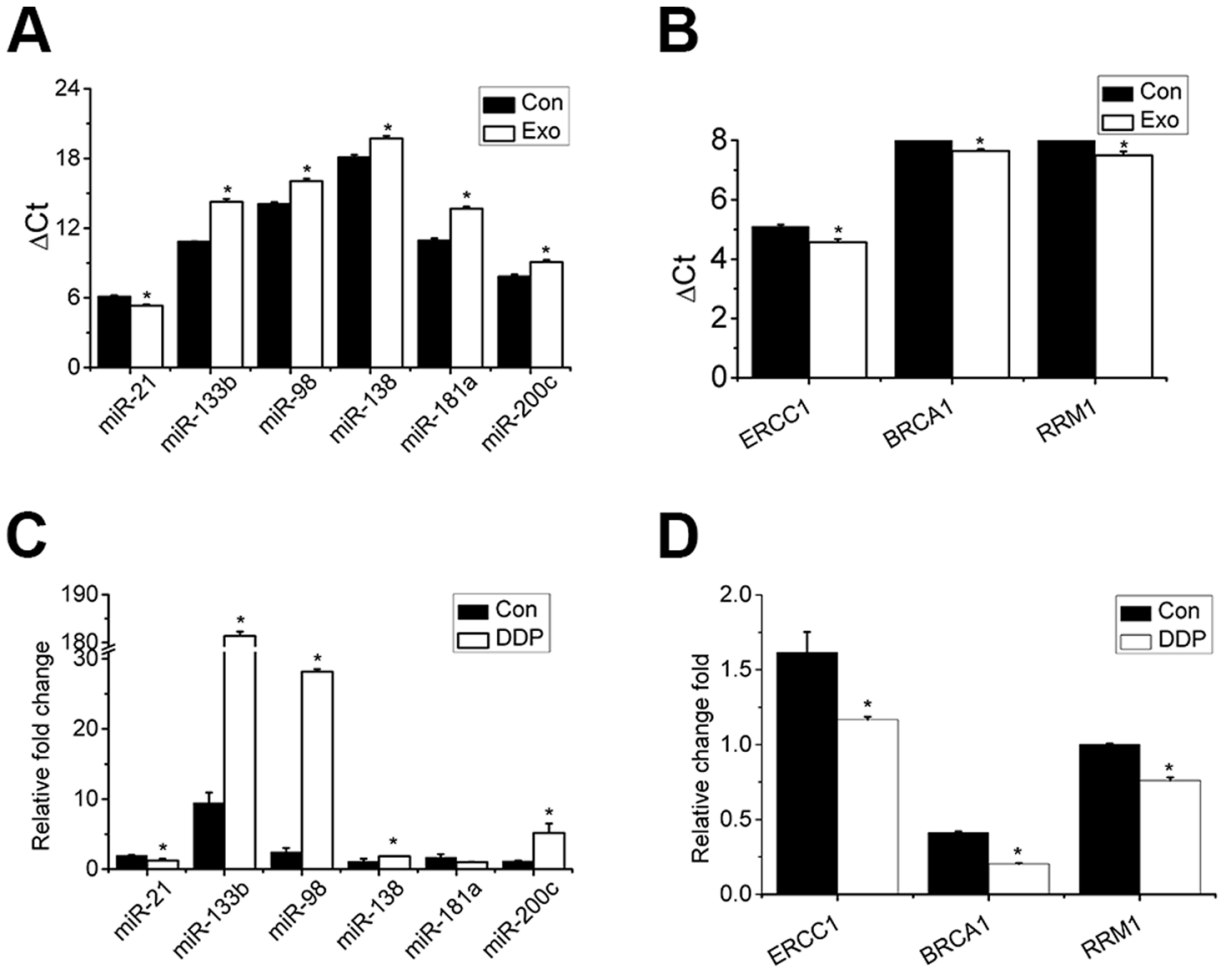
Influence of exosomes on expression of miRNAs and mRNAs detected in this study. Expression levels of (A) miRNAs and (B) mRNAs under normal conditions with and without exosome pretreatment. Fold-changes in expression of (C) miRNAs and (D) mRNAs under cisplatin with and without exosome pretreatment. Bars indicate mean ± SD of three replicates. *indicates p<0.05 versus control groups.

To test possible alternations in expressions of miRNAs and mRNAs in cells as a result of exosomes released by A549 cells during DDP exposure, the expression levels of these miRNAs and mRNAs were detected after cells were exposed to exosomes. As shown in Figure 4A, the expression level of miR-21 increased while the expression levels of miR-98, 133b, 138, 181a, and 200c decreased. Figure 4B shows an increase in the expression levels of ERCC1, BRCA1, and RRM1. Furthermore, as shown in Figures 4C and 4D, after A549 cells were treated with DDP, the relative fold-changes of these miRNAs and mRNAs in cells pretreated with exosomes varied from the relative fold-changes observed in cells cultured under normal conditions.

## Discussion

To the best of our knowledge, our study is the first to demonstrate exosomes involvement in the regulation of sensitivity of lung cancer A549 cells to DDP. We found that exposure of A549 cells to DDP could cause cells to release more exosomes than in normal conditions and that the interaction of these exosomes with other A549 cells could increase the resistance of these A549 cells to DDP. Our study first proves that when A549 cells are exposed to DDP, the expression levels of several miRNAs and mRNAs reportedly associated with DDP sensitivity change significantly in exosomes and that these changes probably mediate the DDP resistance of A549 cells.

Once exposed to DDP, cancer cells adapt to changes in the environment to survive. DNA damage response (DDR) system and diverse signaling pathways are then induced. Some cells can repair this damage and pass through cell cycle checkpoints, whereas other cells are unable to repair the lesions and thus undergo cell death by apoptosis or cellular senescence. As shown in Figure 2A, approximately half of the cells died upon exposure of A549 cells to 3 µg/mL DDP, while the contents and cellular processes of surviving cells were altered at the transcriptional level. As shown in Figure 2E, the expression levels of miR-21, miR-133b, miR-98 and miR-181a detected in this paper varied. RNA contents in exosomes reportedly differ from the RNA of donor cells [10], and the differential expressions of exosomal miRNAs depend on both the origin of cells and the environment. Figure 2G shows that under DDP treatment, the differential expression levels of miRNAs in exosomes differed from those in cells. And as shown in Figure 2E and 2F, cellular miRNAs expressions were different from exosomal miRNAs expressions. Additionally, the expression levels of miRNAs varied in cells grown under different conditions, which indicates that miRNAs contents in exosomes can be regulated according on the biological state of the cells.

During DDP exposure, exosomes released by each surviving cell increase and some of the DDP is exported by these exosomes [16]. Exosomal miRNAs are then mediated in cell-to-cell communication [10]. Figure 3B, 4A, and 4B show that exosomes carrying messages could be uptaken by surrounding cells and cellular contents are altered accordingly. These messages may contain information of imminent danger, and the content in exosomes always depends on the condition wherein the exosomes are released. When cells were treated with a second cycle of DDP, the transcriptional levels were influenced. As shown in Figure 4C, and 4D, cells pretreated with exosomes demonstrated some preparation for the second cycle of DDP treatment and transcriptional levels induced in cells differed.

When exposed to DDP, A549 cells were stimulated and the expression level of miR-21 increased. Because the expression level of miR-21 in exosomes also increased significantly and exosomes could be uptaken by surrounding cells, we inferred that exosomes could mediate more miR-21 to other cells. In fact, an increase in the expression level of miR-21 in other cells was observed, which suggests that the upregulated expression of miR-21 [19] decreases the sensitivity of lung cancer cells to DDP. When exposed to DDP once more, cells received a second stimulation and responded less strongly. The expression levels of miR-21 increased but not as significantly as in the first change. The expression level of miR-133b also increased after A549 cells were treated with DDP. Exosomes mediating miR-133b might be uptaken by surrounding cells and influence the transcriptional levels of other cells. As to abundant of external miR-133b might be mediated into the cells, the expression of miR-133b in cells might be fed back to decrease. The downregulated expression of miR-133b [28] has been shown to decrease the sensitivity of lung cancer cells to DDP. After a second cycle of DDP treatment, cells with poor expression of miR-133b responded and the expression level of miR-133b evidently increased.

Many researchers have expressed various views on the function of exosomes in influencing cell responses to stimulation, and these functions of exosomes have always depended on the type of stimulation. Maria Eldh et al. argued that exosomes influence the response of recipient cells to an external stress stimulus by mediating RNAs from one cell to another [29]. Claire Corcoran et al. found that exosomes partly contribute to cellular communication, resulting in mediating docetaxel-resistance to secondary cells [15]. Our study further demonstrated exosome involvement in the regulation of the sensitivity of A549 cells to DDP during DDP exposure and that this influence is probably regulated by exosomes. However, several inadequacies and shortcomings exist in this work, and the detailed mechanism of DDP resistance will be further researched in our next study.

In this study, we demonstrated that exosomes released by A549 cells exposed to DDP could communicate with other cells and influence the resistance of these cells to DDP. Although detailed mechanism remains unclear, we believe that inhibition of exosome formation and release might present a novel strategy for the future treatment of lung cancer.

## Materials and Methods

### Cell Culture

Cells from the well-characterized human lung adenocarcinoma cancer cell line A549 (Shanghai Cellular Research Institute, China) were maintained in RPMI-1640 medium (Nanjing Kaiji Biology, China) supplemented with 10% FBS (fetal bovine serum, Hyclone Laboratories, USA) at 37°C and under 5% CO_2_. Cells were inoculated in a 96-well plate at a cell density of 6×10^3^ cells/well, and dose-response evaluation of DDP to A549 cells was performed using a cell counting kit-8 (CCK-8; Dojindo, Kumamoto, Japan).

### Isolation and Characterization of Exosomes Released by A549 Cells

To reduce the influence of exosomes in FBS, FBS was depleted of exosomes by ultracentrifugation at 200,000×g at 4°C for 16 h (Beckman 70 Ti rotor). After allowing cells to attach overnight, the medium was replaced with RPMI-1640 medium and 10% exosome-depleted FBS. After 48 h, the culture medium was harvested to collect exosomes. Exosomes isolation (Figure 1A) was performed using a Beckman ultracentrifuge (70 Ti rotor) and ExoQuick-TC exosome precipitation solution (System Biosciences, Mountain View, CA, USA).

Exosomes dissolved in 200 µL of phosphate-buffered saline (PBS) were quantified using an enhanced BCA protein assay kit (Beyotime Biotechnology, China). The morphology and particle size of exosomes dissolved in PBS were characterized via a transmission electron microscope (TEM, JEM-2100, JEOL, Tokyo, Japan). Total cellular and exosomal proteins were respectively extracted from cells and exosomes using SDS lysis buffer (250 nM Tris-HCL, pH 7.4, 2.5% SDS). Proteins (10 mg/mL) were separated on 10% SDS-PAGE gels and transferred to a PVDF membrane. The antibodies used for immunoblotting included CD63 (System Biosciences, Mountain View, CA, USA) and β-actin (Sigma-Aldrich). Using enhanced chemiluminescence solution (ECL kit, Pierce), protein bands were observed using Molecular Image ChemiDoc XRS+ (Bio-Rad).

### Uptake of Exosomes and Cell Viability Analysis

The exosome pellets were resuspended in 1 mL of phosphatebuttered saline (PBS) containing 5 µg/mL DiD (Biotium, USA) for 30 min. The resuspended solution was centrifuged at 200,000×g for 2 h, and the PBS-resuspended pellets were added to A549 cells. Fluorescence imaging was subsequently performed by confocal microscopy.

To assess the effect of exosomes on the sensitivity of cells to DDP, 6×10^3^ cells/well were seeded in a 96-well plate with RPMI-1640 containing 10% exosome-depleted FBS. After 24 h, exosomes released by A549 cells under normal and DDP-treated conditions were added to the corresponding cells at a ratio of 5: 1 (exosomes harvested from 5 mL of supernatant were added into cells cultured in 1 mL of culture medium) for 3 h. The same volume of PBS without exosomes was added to cells used as the control group. For cytotoxicity assays, 3 µg/mL DDP was applied to the wells for 48 h and the total cell viability was assessed using a cell counting kit-8 (CCK-8).

### RNA Isolation and Analysis

Exosomal RNAs were isolated using a mirVana microRNA isolation kit (Life Technologies, Carlsbad, CA, USA) and eluted into 100 µL of heated elution solution according to the manufacturer’s protocol. Cellular RNAs were obtained using Trizol® extraction methodology (Invitrogen, Paisley, UK) and eluted in 30 µL of ddH_2_O.

MiRNA was stem-loop reverse transcribed (RT) with the miRNA reverse transcription kit (Applied Biosystems, Foster City, CA, USA). RT-PCR was performed in triplicate using TaqMan miRNA assay (Applied Biosystems). A gene-specific probe mix (miR-21, 000397; miR-98, 000577; miR-133b, 002247; miR-138, 002284; miR-181a, 000480; miR-200c, 002300) was used in this study. U6 snRNA was used as the internal control for TaqMan miRNA assay to detect the expression level of miRNAs in cells; synthetic non-human miRNA [*Caenorbabditis elegans* miR-39 miRNA (cel-miR-39); Takara Bio Inc., Tokyo, Japan] was spiked in to normalize the exosome volume at the onset of RNA isolation.

Custom TaqMan Low Density RT-PCR array (Confinder Bio., China) was applied to detect mRNA expression, and GAPDH was selected as the internal control. Analysis of gene expression was performed using ABI Prism 7900 Sequence D internal detection system software (Applied Biosystems).

### Statistical Analysis

Results are expressed as mean ± SD. Statistical analysis was performed using Student’s *t*-tests when comparing two groups (PASW Statistics 18.0), and p<0.05 was considered statistically significant.
